# Obesity-related differences in amygdala and hippocampal volume and metabolism before and after a placebo-controlled antidepressant trial in major depressive disorder

**DOI:** 10.1038/s41598-026-43078-7

**Published:** 2026-03-12

**Authors:** Karen Lin, Karin Hasegawa, Vindhya Rapelli, Jie Yang, Ramin V. Parsey, Christine DeLorenzo

**Affiliations:** 1 Center for Neurointestinal Health, Massachusetts General Hospital, Boston, MA USA; 2 Division of Nutritional Sciences, Cornell University, Ithaca, NY USA; 3 Department of Applied Mathematics and Statistics, Stony Brook University, New York, NY USA; 4 Stony Brook University, New York, NY USA; 5 Department of Family, Population & Preventive Medicine, Stony Brook University, New York, NY USA; 6 Department of Psychiatry and Behavioral Health, Stony Brook University, Stony Brook, NY USA; 7 Department of Biomedical Engineering, Stony Brook University, Stony Brook, NY USA

**Keywords:** Diseases, Medical research, Neuroscience

## Abstract

This study examined the association between obesity, measured by waist circumference (WC) and Body Mass Index (BMI), and neurobiology of the treatment/placebo response in depression. 85 participants (56 females, 29 males) with Major Depressive Disorder (MDD) were imaged using PET/MRI before and after a double-blind, randomized escitalopram trial. Analyses were pooled across interventions to investigate primary imaging associations. Linear mixed models examined associations between obesity measures and amygdala and hippocampal volume and metabolism. Preintervention obesity was significantly positively associated with amygdala and hippocampus volume, but not metabolism. Successful treatment is often associated with an *increase* in volume and a *reduction* in metabolism in these regions. However, greater WC and BMI were associated with *decreases* in amygdala volume following treatment/placebo. And greater BMI was associated with *increases* in amygdala and hippocampus metabolism following treatment/placebo. Importantly, however, these neurobiological changes were not associated with differential improvement in depression severity. Overall, this multimodal study shows obesity-related factors are associated with intervention-related neurobiological changes in MDD; however, additional research is needed to clarify whether these alterations correspond to meaningful variation in symptom improvement.

## Introduction

According to the World Health Organization (WHO), depression is the most common mental illness, affecting 280 million individuals worldwide^1^. Contributing to depression’s impact, there is broad variation in antidepressant therapy response,^2^leading to unresolved depression, impairing an individual’s quality of life and heightening mortality^3^. One factor that has been associated with poor treatment outcomes is obesity^2^. For example, obese individuals demonstrate unfavorable treatment outcomes including resistance to selective serotonin reuptake inhibitors (SSRIs)^4–6^..

The amygdala and hippocampus are implicated in the biology of major depressive disorder (MDD), in antidepressant treatment response, and in obesity^7^, and therefore may be involved in associations between obesity and response to treatment in MDD. Numerous studies report smaller hippocampal volume in depression^8–17^and generally smaller amygdala volumes^18–21^, though transient enlargement of the amygdala during the first episode of depression may occur^22,23^with decreased volume in subsequent years^24^. Functionally, greater depression severity has been associated with lower hippocampal metabolism^25^and elevated amygdala activity^26–30^..

Comparable patterns are observed in obesity, with smaller hippocampal volume^31–34^and mixed findings for the amygdala – some studies show reduced gray matter^35,36^, while others link greater adiposity^37^ or poor dietary impulse control^38^to larger amygdala volumes. Nonetheless, diets high in fat or ultra-processed foods are consistently associated with reduced hippocampal and amygdala volumes^39,40^. Functionally, greater visceral adiposity and future weight gain is associated with increased amygdala activity^41^. Hippocampal metabolism may also be elevated in obesity, as suggested by diminished hippocampal glucose utilization following bariatric surgery^42^..

Altered brain volumes and activity in both depressed and obese individuals may be attributed to numerous factors, including altered hypothalamic-pituitary-adrenal (HPA) axis function. HPA axis activity could be influenced through multiple pathways, including inflammation, reported in both depression^43–45^ and obesity^46,47^, increased glucocorticoids, potentially through increased stressful life events reported in depression^48,49^ or increased adipose tissue in obesity^50^, and reduced brain-derived neurotrophic factor (BDNF) which is lower in depression^51^and obesity^52,53^..

Antidepressants have been shown to increase hippocampus and amygdala volume^21,54^and decrease amygdala and hippocampus activation^28,55–57^. These changes have been associated with antidepressant response^58,59^. However the neurobiological mechanisms that have been proposed to underlie treatment-related changes -- normalizing HPA activity, lowering glucocorticoid levels^60^, or increasing production of BDNF, supporting neurogenesis in the hippocampus^61,62^ and amygdala^21^– may be associated with obesity level.

We therefore examine: (1) the relationship between obesity and intervention outcome. We hypothesize that greater preintervention waist circumference will be associated with reduced depression improvement following intervention, pooling escitalopram and placebo arms.

To examine potential neurobiological mechanisms, we assess: (2) the association between waist circumference and amygdala and hippocampal volume and metabolism before intervention. And, (3) the association between waist circumference and amygdala and hippocampal volume and metabolism changes with intervention, pooling escitalopram and placebo arms. Although metabolic changes can occur more rapidly than structural changes^63,64^, both have been reported with antidepressant treatment and each may be associated with treatment response; therefore both are examined in this work.

We hypothesize that, in depressed individuals, preintervention waist circumference is negatively associated with hippocampus and amygdala volume and positively associated with amygdala and hippocampus metabolism. As the effects of obesity may be associated with neurobiological response to treatment, we further hypothesize that greater preintervention waist circumference will be associated with a smaller percent increase in hippocampus and amygdala volume and a smaller reduction in amygdala and hippocampus metabolism following intervention. Our primary analyses use continuous measures of waist circumference; however, to further contextualize these findings, we also examined discrete waist circumference categories defined by metabolic risk.

Obesity is typically assessed through body mass index (BMI). However abdominal obesity, also known as central or visceral obesity, assessed by waist circumference, has shown a stronger association with depression than BMI^65,66^. This is possibly due to differences in the type of adipose tissue near the abdomen. White adipose tissue (WAT) can be divided into two subdivisions, visceral or subcutaneous, with distinct structural and metabolic characteristics^67^. Visceral WAT exhibits greater metabolic activity, produces higher systemic inflammation and has direct access to the portal circulation, unlike subcutaneous WAT^65,68^. In addition to depression, the accumulation of visceral WAT is associated with metabolic disorders, including dyslipidemia, hypertension and insulin resistance, and cardiovascular disease^67,69^. Therefore, waist circumference is used as the primary assessment of obesity in this study. However, following the 2025 Lancet Commission recommendation to use multiple anthropometric measures^70^, we also consider BMI in exploratory analyses.

## Methods

The study “Advancing Personalized Antidepressant Treatment Using PET/MRI” is registered on ClinicalTrials.gov (NCT02623205, date of registration is 01/05/2015) and approved by the Institutional Review Board of Stony Brook University (see CONSORT diagram in^71^).

### Participants

All participants were assessed via the Structured Clinical Interview for DSM-IV (SCID-IV) and were verified by a trained rater as having been diagnosed with MDD, in a current major depressive episode, with a score of 22 or higher on the Montgomery–Åsberg Depression Rating Scale^72^(MADRS). Inclusion criteria consisted of: 18 years old or older and the ability to sign informed consent. All participants provided written informed consent prior to participation, and all procedures were conducted in accordance with relevant guidelines and regulations. Potential participants were excluded if they had a lifetime history of bipolar disorder, a significant active physical illness (including metabolic disorder), significant neurological deficits, current psychosis, or a high potential for excessive substance use during the study period (including substance/alcohol dependence within 6 months and abuse within two months, excluding nicotine). Additional exclusion criteria included participants receiving successful antidepressant treatment, who had electroconvulsive therapy within six months, medical contraindications to escitalopram (study drug), such as failed escitalopram therapy of appropriate dose and duration in the past, or contraindications to MRI or positron emission tomography (PET) imaging, such as pregnancy or breastfeeding.

Data from this cohort has been previously examined, to predict intervention response from preintervention measures^71,73–75^, to examine functional changes with intervention^71,76,77^, effects of intervention on brain structure^77^, as well as the association between neurobiology and fatigue^78^ social anhedonia^79^ and childhood trauma^80^. None have examined obesity.

### Study design

Height, weight and waist circumference were measured at the participant screening visit. All participants were antidepressant medication-free for three weeks prior to the study. For those who entered the study on ineffective medication, washout was completed over a maximum of four weeks before the three-week psychotropic medication free period. Simultaneous PET/MRI imaging was acquired on a 3 T Siemens Biograph mMR (Siemens, Erlangen, Germany) with a 12-channel head coil for 60 min in both the pre and post intervention session (average time between scans: 59.6 ± 8.8 days). The Hamilton Depression Rating Scale (HAM-D-17) was assessed within approximately one week of imaging. Treatment/placebo was initiated following preintervention imaging. Through a double-blind design, participants were randomized to placebo or escitalopram. Analyses were conducted pooling escitalopram and placebo arms, with intervention type included as a covariate.

### Magnetic resonance imaging (MRI)

A magnetization-prepared rapid gradient-echo (MP-RAGE) T1-weighted structural image was acquired, simultaneously with the PET imaging, with the following parameters: TR=2300ms, TE = 3.24ms, flip angle = 9 degrees, IPAT GRAPPA factor 2, FOV=223 × 210 × 195 mm, bandwidth = 220 Hz/Px, echo spacing = 7.8ms, voxel size = 0.87 × 0.87 × 0.87 mm. T1 structural images were processed through the segmentation pipeline of Freesurfer 5.3.0 (http://surfer.nmr.mgh.harvard.edu*)*to automatically extract the amygdala and hippocampus from the Desikan-Killiany atlas^81,82^..

### Positron emission tomography (PET)

Up to 185 MBq of 2-[^18^F]-fluorodeoxyglucose (FDG) were injected intravenously and emission data was acquired for 60 min on a Siemens Biograph mMR. Raw listmode PET data were reconstructed offline using Siemens’ e7 Tools software and a CT-like Boson MR-based attenuation map^83,84^. Frames were corrected for motion^85^and co-registered to the MRI. The regional delineations were transferred to the PET images through the co-registration. Metabolic rate of glucose uptake (MRGlu) was estimated from the Patlak graphical using the time activity curve while correcting for blood glucose and the lumped constant, using Simultaneous Estimation and a single venous sample, as previously described^71^..

### Statistical analysis

As described above, this study examines: (1) the relationship between preintervention waist circumference and intervention (escitalopram/placebo) outcome, (2) the association between preintervention waist circumference and amygdala and hippocampal volume and metabolism before intervention, and (3) the association between waist circumference and amygdala and hippocampal volume and metabolism changes with intervention.

In a prior study of this cohort, neurobiological changes during the intervention period, quantified by FDG-PET, were not different across interventions (escitalopram/placebo)^71^,in accordance with a previous study of brain metabolism, in which consistent metabolic changes were observed in depressed participants in response to fluoxetine and placebo^86^. It has been hypothesized that this common brain biological response may be due to placebo-induced activation of neurotransmission^87^, and could explain the similar response rates^88–90^ between SSRI and placebo. For these reasons, the escitalopram and placebo subgroups were examined together, with intervention type included as a covariate.

Model 1: Multiple linear regression models were utilized to examine the relationship between preintervention waist circumference and Hamilton Depression Rating Scale score as well as change in this score with treatment/placebo after controlling for sex and age.

Here, preintervention waist circumference was treated as a predictor, and depression score, or change in depression score, was treated as an outcome in the models.

Model 2: Linear mixed models were used to examine the association between continuous values of preintervention waist circumference and:

- preintervention amygdala and hippocampus volume after controlling for sex, age, age^2^(to account for nonlinear effects) and preintervention total brain volume,
- preintervention amygdala and hippocampus metabolism after controlling for sex, age, and age^2^.

Here, preintervention waist circumference was treated as a predictor, and preintervention brain measure was treated as an outcome in the models.

To shed light on Model 2 results, linear mixed models were also used to examine differences in the variables listed above across discrete levels of waist circumference, grouped by metabolic risk. For this analysis, participants were grouped into three categories: low risk (≤ 94 cm for males and ≤ 80 cm for females), increased risk (> 94 to 102 cm for males and > 80 to 88 cm for females) and substantially increased risk (> 102 cm for males and > 88 cm for females)^91^..

Model 3: Similar linear mixed models were used to examine the association between preintervention waist circumference and:

- percent change in amygdala or hippocampal volume, controlling for intervention (escitalopram/placebo) type.
-  percent change in amygdala or hippocampal metabolism, controlling for intervention (escitalopram/placebo) type.

Here, preintervention waist circumference was treated as a predictor, and percent change in the brain measure was treated as an outcome in the models.

Percent difference, calculated using postintervention value minus preintervention value divided by preintervention value, was used as an outcome in order to normalize post-intervention outcomes to each individual’s preintervention values, be consistent with the study hypotheses, which focus on relative change from preintervention values rather than absolute differences with intervention and because, with only two timepoints (pre and post), modeling timepoint as a factor is often equivalent to analyzing change scores.

To shed light on Model 3 results, linear mixed models were also used to examine differences in the variables listed above across discrete levels of waist circumference, grouped by metabolic risk.

As an exploratory analysis, Models 1–3 were repeated with BMI instead of waist circumference. For this analysis, participants were grouped into four categories: underweight (< 18.5 lb/in^2^), healthy weight (18.5 to 25 lb/in^2^), overweight (25 to 30 lb/in^2^) and obese (> 30 lb/in^2^)^92^.

For each linear mixed model, a covariance structure was utilized to model the correlated measures from the amygdala and hippocampus. Final dependence structure was chosen from Compound Symmetry and Unstructured based on the Akaike Information Criteria (AIC). For each result, p-values from Type III T-test for testing the significance of the pairwise difference (noted with a single asterisk in Tables 4 and 5), and p-values from Type III F-test for testing the significance of an interaction term (noted with a double asterisk in Tables 4 and 5) were reported in addition to the estimated coefficients or difference and 95% confidence intervals.

**Table 1:** Preintervention participant demographics.

Variable	*N*	Levels	Total
Sex	85	Female	56 (65.88%)
		Male	29 (34.12%)
Age	85		23.60 ± 13.10
Medication Status	85	Naïve	45 (52.94%)
		Previously medicated	40 (47.06%)
BMI (lb/in^2^)	84		25.45 ± 8.13
BMI Category	84	Underweight	5 (5.95%)
		Healthy weight	34 (40.48%)
		Overweight	22 (26.19%)
		Obese	23 (27.38%)
Waist circumference (in)	76		34.00 ± 7.00
Waist circumference Category	76	Low risk	35 (46.05%)
		Increased risk	14 (18.42%)
		Substantially increased risk	27 (35.53%)

Note: For categorical variables, percentages across the levels were reported. For continuous variables, median and interquartile range were reported.

**Table 2:** Participant depression severity and brain measures before and after placebo-controlled intervention. analyses were conducted pooling escitalopram and placebo arms. AMY: amygdala; BMI: body mass index; HIP: hippocampus.

Variable	*N*	Levels	Total
*Hamilton Depression Rating Scale (HAM-D) Scores*			
Preintervention HAM-D Score	85	18.00 ± 5.00	
Postintervention HAM-D Score	78	10.00 ± 9.00	
% Change in HAM-D Score	78	−44.44 ± 44.59	
*Preintervention Brain Measures*			
Preintervention total AMY volume (mm^3^)	85	3183.20 ± 542.50	
Preintervention total HIP volume (mm^3^)	84	8494.30 ± 1100.35	
Preintervention total AMY metabolism (mg/min*100 ml)	73	3.14 ± 0.66	
Preintervention total HIP metabolism (mg/min*100 ml)	73	3.49 ± 0.69	
*Postintervention Brain Measures*			
Postintervention total AMY volume (mm^3^)	76	3227.00 ± 482.00	
Postintervention total HIP volume (mm^3^)	76	8517.00 ± 1044.00	
Postintervention total AMY metabolism (mg/min*100 ml)	72	3.06 ± 0.55	
Postintervention total HIP metabolism (mg/min*100 ml)	72	3.34 ± 0.63	
*% Change in Brain Measures*			
% Change in AMY Volume	76	0.92 ± 9.19	
% Change in HIP Volume	75	−0.49 ± 3.61	
% Change in AMY Metabolism	62	−1.26 ± 22.46	
% Change in HIP Metabolism	62	−3.76 ± 22.09	

Note: For continuous variables, median and interquartile range were reported.

## Results

Of the 85 participants who received preintervention imaging, 12 participants’ PET measures were not used because they exhibited a > 20% change in glucose during the preintervention imaging (*n* = 11) or due to diagnosis of diabetes (*n* = 1, removed from all PET analyses). The Freesurfer delineation of the hippocampus from one participant’s preintervention MRI was misaligned and therefore was not used in the volumetric analysis. Additionally, 12 participants’ postintervention PET measures were not used because the participants did not complete the protocol (*n* = 7), or complete postintervention imaging (*n* = 2, 1 completed MRI only) or they exhibited a > 20% change in glucose during the postintervention PET imaging (*n* = 3). (Note: as one participant exhibited a > 20% change in glucose during the imaging both pre and post intervention, this left 62 participants with useable pre-and post-PET imaging.) Of the 77 participants receiving postintervention MRI imaging, one participant’s MRI measures could not be used due to excessive motion. In addition, 9 participants had missing waist circumference measurements, and one participant had missing weight information preventing BMI calculations. For three participants, self-reported weight was used instead of that measured at screening.

Participant information is provided in Tables 1 and 2.

**Table 3:**
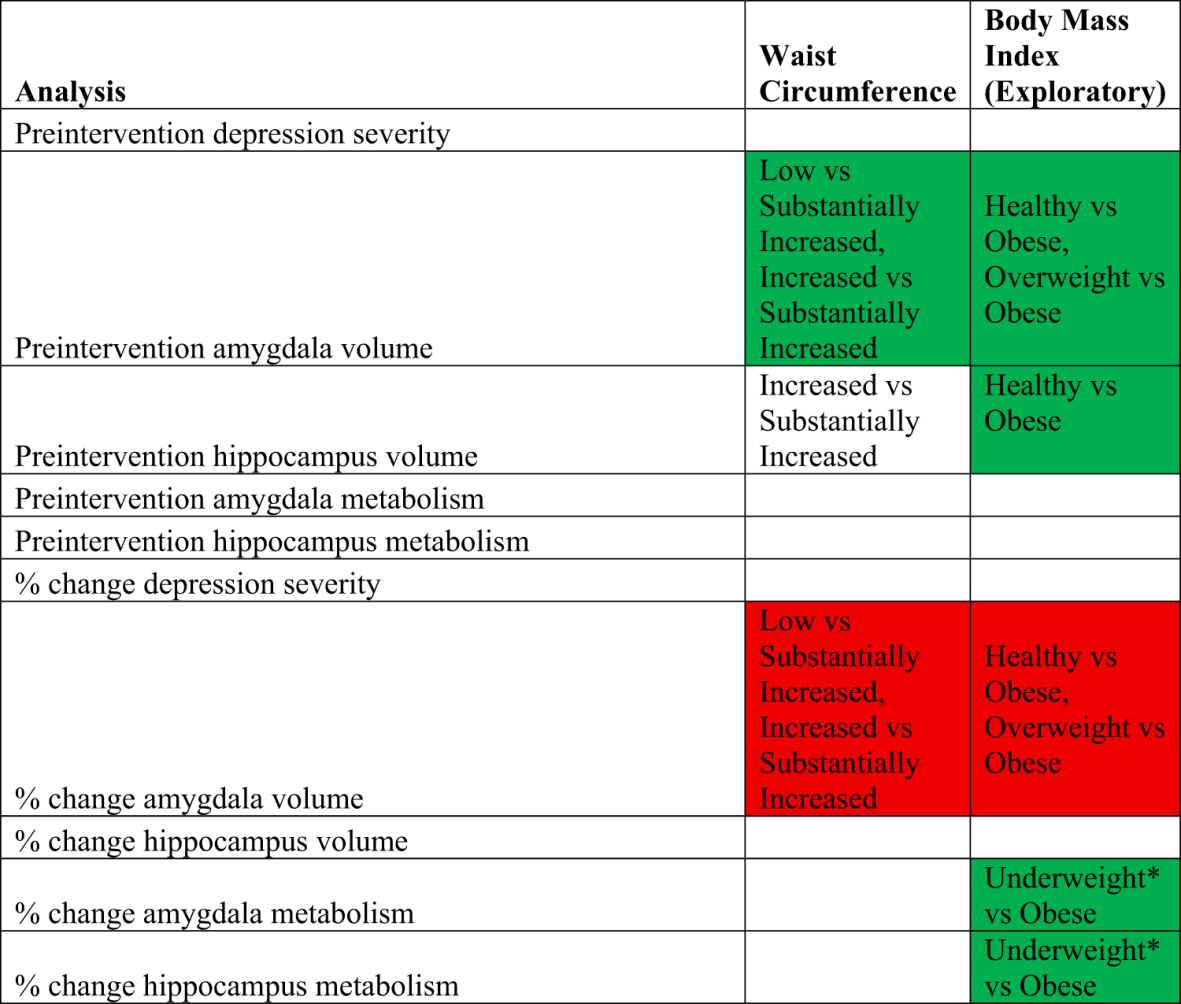
Visualization of study results. The first column indicates the outcome variable examined. Depression severity was assessed by the Hamilton Depression Rating Scale. The colored cells indicate that the outcome variable was significantly related to the continuous measure of depression severity, waist circumference or body mass index. Green indicates a positive association while red indicates a negative association. The text within the cells in columns 2 and 3 specifies the categorical comparisons that reached significance. Blank cells indicate no significant continuous or categorical differences. * These results should be interpreted with caution, as only four participants classified as underweight had usable pre- and post-intervention metabolic data.

**Table 4:**
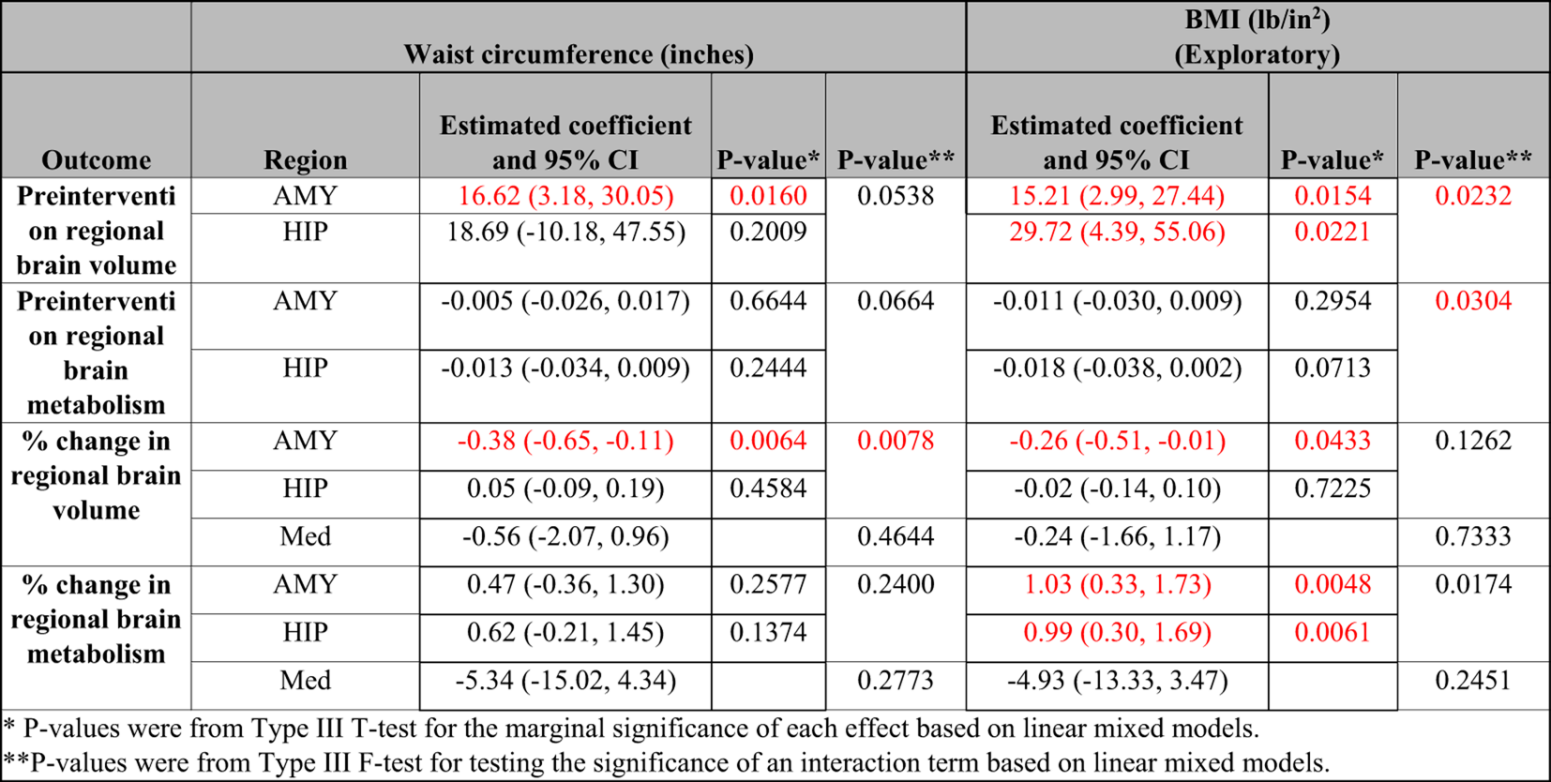
Associations between obesity and brain measures. AMY: amygdala; BMI: body mass index; HIP: hippocampus; Med: Medication --examining whether outcome differed in those receiving escitalopram vs. placebo.

### Visualization of study results

For ease of reference, a visualization of study results is provided in Table 3. These results are explained in subsequent sections.

Relationship between Waist Circumference or BMI and Depression Severity.

As indicated by the blank cells in Table 3, neither preintervention waist circumference (p-value = 0.8471) nor BMI (p-value = 0.1144) was significantly associated with preintervention Hamilton Depression Rating Scale (HAM-D) scores. Similarly, neither preintervention waist circumference (p-value = 0.6640) nor BMI (p-value = 0.1793) was significantly associated with percent change in HAM-D scores with treatment/placebo.

**Table 5:**
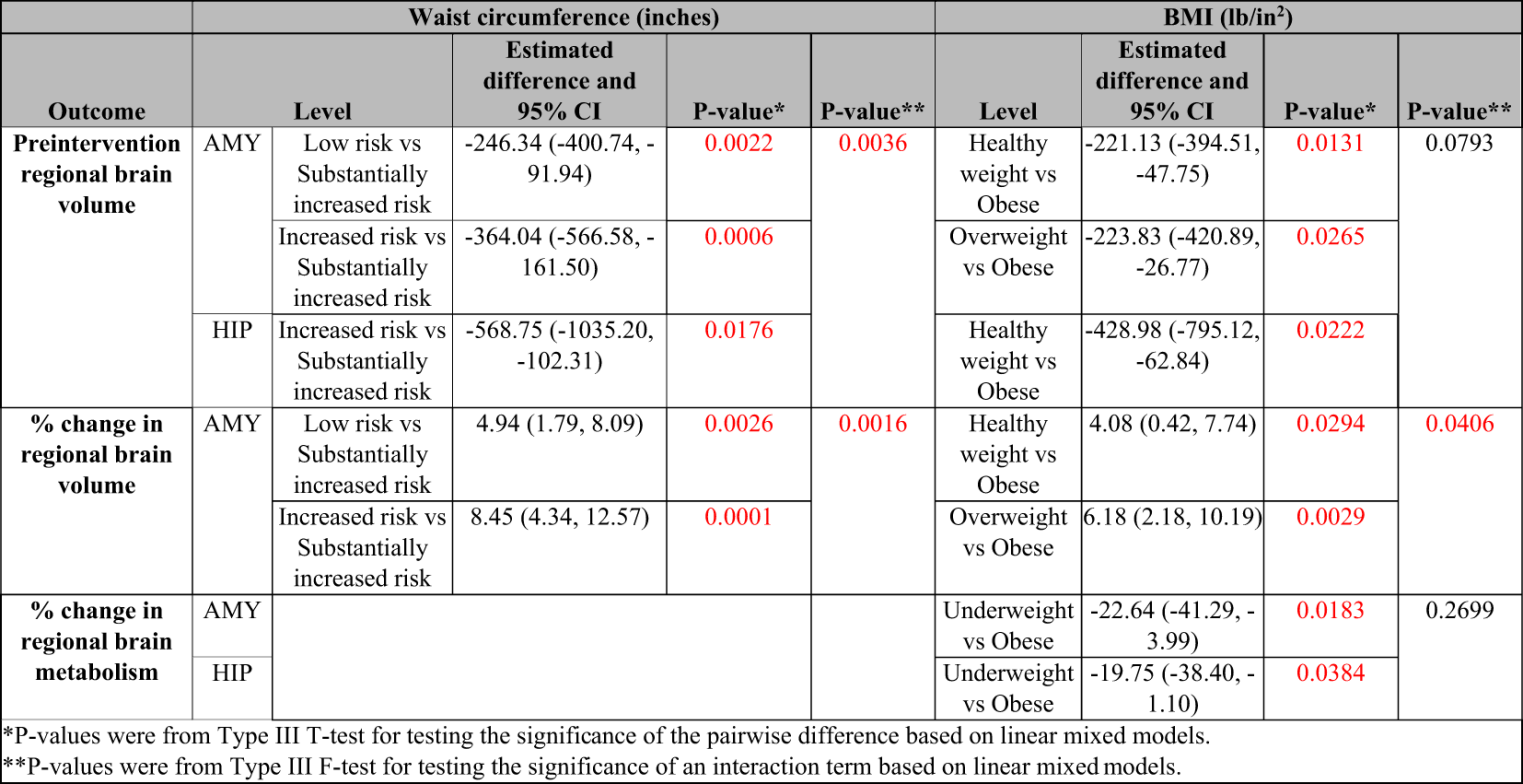
Significant associations between categories of obesity and brain measures. AMY: amygdala; BMI: body mass index; HIP: hippocampus.

A p-value less than 0.025 was considered as statistically significant in order to account for the two models considering neurobiological variables (Models 2 and 3). No correction was applied to the exploratory analyses. All the statistical analyses were performed using SAS 9.4 (SAS Institute Inc., Cary, NC).

There were also no significant differences in preintervention HAM-D scores or percent change in HAM-D scores across waist circumference risk categories or BMI categories (all p-values > 0.05).

There was no effect of intervention type on any of these associations.

Relationship between Waist Circumference or BMI and Preintervention Brain Volume.

A significant positive association was found between preintervention waist circumference and preintervention amygdala volume (Table 4, p-value = 0.016) but not with preintervention hippocampus volume (Table 4).

The discrete analysis provides additional insight into these associations, showing preintervention amygdala and hippocampus volumes were significantly different across some preintervention waist circumference categories (Table 5: amygdala: low vs. substantially increased risk: p-value = 0.002; increased vs. substantially increased risk: p-value = 0.001; hippocampus: increased risk vs. substantially increased risk: p-value = 0.018).

In the uncorrected exploratory analysis, significant positive associations were found between preintervention BMI and both preintervention amygdala (Table 4, p-value = 0.015) and hippocampus (Table 4, p-value = 0.022) volumes.

Similar to the continuous analysis, preintervention amygdala and hippocampus volumes were significantly different across some preintervention BMI categories (Table 5: amygdala: healthy vs. obese: p-value = 0.013; overweight vs. obese: p-value = 0.027; hippocampus: healthy vs. obese: p-value = 0.022).

Relationship between Waist Circumference or BMI and Preintervention Brain Metabolism.

Neither preintervention waist circumference nor BMI were significantly associated with preintervention amygdala or hippocampus metabolism (Table 4).

Similar to the continuous analysis, there were no significant differences in preintervention amygdala and hippocampus metabolism across waist circumference or BMI categories.

Relationship between Waist Circumference or BMI and Percent Change in Brain Volume with Treatment/Placebo.

**Fig. 1:**
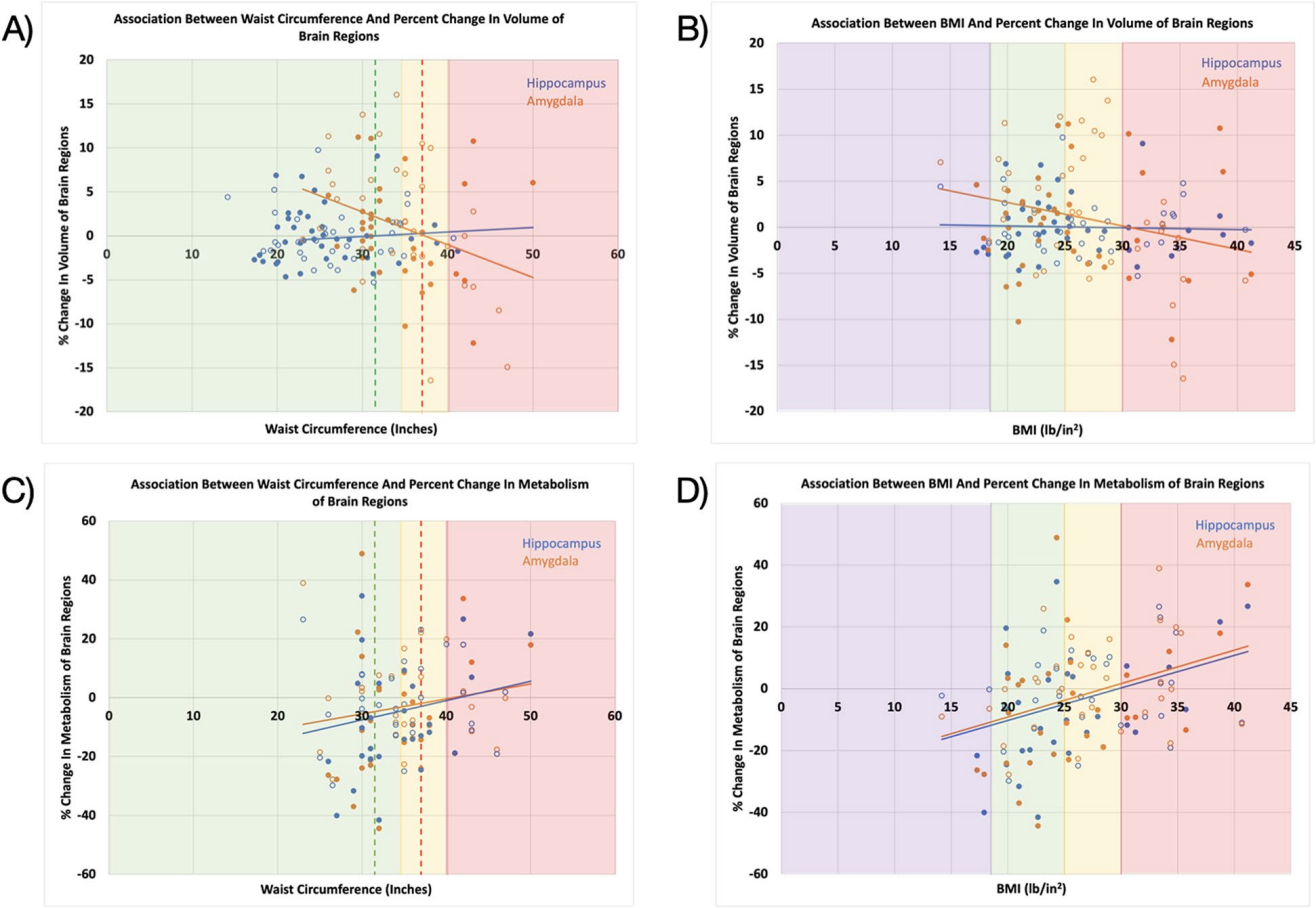
Associations Between Waist Circumference (**A** and **C**) and Body Mass Index (BMI, **B** and **D**) and Percent Change in Brain Region Volume (**A** and **B**) and Metabolism (**C** and **D**) with intervention. Raw values are presented. Solid circles indicate escitalopram treatment and open circles indicate placebo. Regression lines are shown for each brain region. In A and C, based on waist circumference values in males: red- substantially increased risk; yellow – increased risk; green: low risk. The dashed line denotes the cutoff for females: red - between increased and substantially increased risk; green- denotes the cutoff between low and increased risk. In B and D, based on BMI: red – obese; yellow – overweight; green: healthy weight; lavender- underweight.

A significant negative association was found between preintervention waist circumference and the percent change in amygdala volume with treatment/placebo (Table 4; Fig. 1A, p-value = 0.006) but not with percent change in hippocampus volume with treatment/placebo (Table 4; Fig. 1A).

The discrete analysis provides additional insight into these associations showing percent change in amygdala volume was significantly different across preintervention waist circumference categories when compared to the substantially increased risk category (Table 5: low vs. substantially increased risk: p-value = 0.003; increased vs. substantially increased risk: p-value = 0.0001). There were no significant differences in percent change in hippocampus volume across waist circumference categories.

Similarly, in the uncorrected exploratory analysis, a significant negative association was found between preintervention BMI and the percent change in amygdala volume with treatment/placebo (Table 4; Fig. 1B, p-value = 0.043) but not with percent change in hippocampus volume with intervention (Table 4; Fig. 1B).

Similar to the continuous analysis, percent change in amygdala volume was significantly different across some preintervention BMI categories when compared to the obese category (Table 5: healthy vs. obese: p-value = 0.029; overweight vs. obese: p-value = 0.003). There were no significant differences in percent change in hippocampus volume across BMI categories.

There was no effect of intervention type on any of these associations (Table 4).

Relationship between Waist Circumference or BMI and Percent Change in Metabolism with Intervention.

Preintervention waist circumference was not significantly associated with percent change in amygdala or hippocampus metabolism with treatment/placebo (Table 4; Fig. 1C).

Similar to the continuous analysis, percent change in amygdala and hippocampus metabolism was also not significantly different across categories of preintervention waist circumference (Table 5).

In the uncorrected exploratory analysis, significant positive associations were found between BMI and both percent change in amygdala (Table 4; Fig. 1D, p-value = 0.005) and hippocampus (Table 4; Fig. 1D, p-value = 0.006) metabolism with treatment/placebo.

The discrete analysis provides additional insight into these associations, showing percent change in metabolism was only significantly different across BMI-defined categories of underweight vs. obese individuals (Table 5: amygdala: p-value = 0.018; hippocampus: p-value = 0.038). Notably, the underweight group included only four participants with usable pre- and post-intervention metabolic data.

There was no effect of intervention type on any of these associations (Table 5).

## Discussion

In this study, two anthropometric measures were used to evaluate body composition or adiposity in relation to neurobiology in individuals with MDD before and after intervention. Waist circumference, measuring visceral fat, is one among five diagnostic criteria for diagnosing metabolic syndrome^93,94^. However, BMI is associated with body fat and a measure that is well-established in the literature for measuring obesity due to its practicality and ease of collection.

While waist circumference and BMI showed similar relationships to brain biology in this work (Table 5), an exception was percent change in metabolism with intervention/placebo, which only differed across exploratory BMI measures. However, analysis treating BMI as a categorical variable provides insight into this association, suggesting it was driven by difference between the underweight participants and the obese participants. Given the size of this subgroup (*n* = 4, Fig. 1D), this finding is hypothesis-generating only, and should be interpreted with caution and confirmed in a larger sample. However, this is consistent with the fact that waist circumference does not categorize underweight individuals (e.g., large green area in Fig. 1A and C compared to Fig. 1B and D) and is generally not a measure of overall obesity, but rather metabolic disease and risk^95–97^..

### Relationship between Waist circumference or BMI and preintervention neurobiology

Contrary to our hypothesis, but consistent with a prior study^37^, a positive association between obesity measures and preintervention amygdala volume was found. Examining preintervention amygdala volume differences across obesity categories in Tables 2 and 5 suggests that this correlation may be driven by individuals with higher waist circumference or BMI.

The association between obesity measures and hippocampal volume was not as strong, suggesting hippocampal differences may only be observable over specific ranges of obesity measures.

Notably, the associations between waist circumference or BMI and brain volume exist even though no association between waist circumference or BMI and depression severity was found, suggesting that depression severity did not confound these relationships.

Neither preintervention waist circumference nor BMI was associated with metabolism (hippocampus or amygdala). This lack of correlation may indicate that baseline metabolism is not as strongly correlated to obesity as changes in metabolism are (described in the next section).

### Treatment/placebo response

Though this study involved both escitalopram and placebo, the percent change in volume and metabolism were not significantly different across interventions (Table 4). Accordingly, and consistent with prior analyses of this cohort^71^, intervention arms were pooled for analyses. However, this approach represents an important limitation (as noted below). Combining escitalopram and placebo groups constrains the ability to draw conclusions about antidepressant-specific mechanisms and may obscure subtle differential drug effects. Moreover, while shared metabolic changes could reflect placebo-related neurobiological processes, this interpretation cannot be definitively established within the current design and should be considered cautiously.

Importantly, consistent with other studies^98,99^, there was significant variability in intervention response across the cohort (for both treatment and placebo, see standard deviation in percent change in the Hamilton Depression Rating Scale, Table 1). Likely relatedly, there was significant variation in the neurobiological response to the interventions (Table 1), which has also been reported^100–103^. The purpose of this study is to understand the association between obesity and this variability.

Despite observing associations between obesity and neurobiological changes with treatment/placebo (described below), we did not observe a significant relationship between obesity and percent change in Hamilton Depression Rating Scale score following intervention. Although brain volumetric and metabolic measures (e.g., amygdala and hippocampal changes) represent direct, quantifiable indices of intervention-related neuroplasticity, whereas clinical symptom scales capture a composite of psychological, social, behavioral, and biological influences^104,105^, the absence of a detectable differential clinical response in this sample limits the immediate translational implications of these findings. Accordingly, while neuroimaging measures may be sensitive to subtle obesity-related variation in brain processes, it remains unclear whether these differences meaningfully influence symptomatic improvement. Future studies, ideally with larger samples and integrated assessments of inflammatory markers, adiposity indices, and neuroimaging measures, will be needed to clarify whether obesity-related biological factors shape clinically relevant intervention mechanisms.

### Relationship between Waist circumference or BMI and percent change in neurobiology with treatment/placebo

Both preintervention waist circumference and BMI showed a significant negative association with percent change in amygdala volume. Every 1 in increase in waist circumference or 1 lb/in^2^ increase in BMI, was associated with a 0.38% or a 0.26%, respectively, *decrease* in percent change in amygdala volume with treatment/placebo. In fact, past 37.29 (Fig. 1A) in, which is the border of increased risk for males and substantially increased risk for females, or 30.63 lb/in^2^ (Fig. 1B), which is the obese range, the regression fit line suggests the percent change in amygdala volume with treatment/placebo becomes negative. When combined with the categorical analyses (examining percent change in amygdala volume across obesity categories), results indicate that intervention-related changes in amygdala volume are associated with obesity severity.

No significant relationship between waist circumference or BMI and percent change in hippocampal volume with treatment/placebo was found. This highlights both that obesity/intervention interactions may be region specific and the importance of the amygdala in this interaction.

When examining the relationship between obesity measures and metabolic changes with treatment/placebo, the percent change in metabolism from negative (expected with successful treatment) to positive occurs at 41.50 in (substantially increased risk) or 29.77 lb/in^2^ (obese range is > 30) for the hippocampus and 40.90 in or 28.46 lb/in^2^ for the amygdala, though the significant relationships were observed only using BMI. Further, every 1 lb/in^2^ increase was associated with a 1.03% or a 0.99% increase in percent change in amygdala or hippocampus metabolism, respectively. Across the range of BMIs in this study (14.2–41.2 lb/in^2^), this would correspond to a significant (27–28%) difference in amygdala or hippocampal metabolism.

### Limitations

While waist circumference and BMI are non-invasive, cost-effective and easy to measure, the cutoffs and categorization of individuals do not sufficiently capture the nuance of individual body composition, especially for individuals at the margin between two categories. The gold standard for assessing body composition, the dual-energy x-ray absorptiometry (DXA), was not utilized in this study and could be a potential limitation.

In addition, diet quality, physical activity, sleep patterns, and stress exposure, all of which are associated with both obesity and brain structure/function, were not quantified in this study. These factors may partially mediate or confound the observed relationships between adiposity and neurobiological outcomes and should be incorporated into future studies to improve model specificity.

It is also important to note that the relatively young median age of participants in this study, 23.6 years, substantially limits the generalizability of the findings to the broader population. As such, these results should not be assumed to extend to midlife or geriatric populations, in whom metabolic burden, illness chronicity, and treatment exposure may interact differently with brain structure and function. However, this younger cohort remains an important group to investigate as the observed obesity-related associations may reflect early stage or developmentally specific features that differ from patterns in later-life depression. Whether modifying obesity at this age would alter treatment-related neurobiological changes or clinical outcomes remains unknown and warrants future investigation.

Importantly, although this study included both escitalopram and placebo arms, the intervention groups were pooled due to the absence of significant between-group differences. This approach precludes conclusions about SSRI-specific mechanisms. Accordingly, the findings should be interpreted as reflecting intervention-associated brain changes in MDD more generally, rather than antidepressant-specific associations. Future studies directly comparing active treatments and placebo, as well as different antidepressant classes, will be necessary to determine the specificity of obesity-related associations with neurobiological changes during treatment.

The inter-individual variability in functional changes with treatment/placebo was high in the sample. As this has been observed in previous studies^100,101^, it is likely reflective of meaningful biological heterogeneity. However, this should be examined in future studies. In addition, as the study only assessed volume and metabolism, it cannot determine the mechanisms underlying differences in these measures.

In this study, approximately half of the participants were previously medicated (Table 1), which could influence preintervention brain volume or metabolism, as well as intervention-related changes in these measures. When prior medication exposure (exposed vs. naïve) was included as a covariate (data not shown), the interpretation of the results did not change materially. However, inclusion of more detailed measures of prior medication exposure (e.g., timing and class) as covariates may be informative.

## Conclusion

This multimodal PET/MRI study found that obesity is associated with altered limbic structure and metabolism in MDD, both prior to and during treatment/placebo. While greater obesity was linked to larger preintervention amygdala volume and altered intervention-related changes in amygdala volume and metabolism, no significant association was observed between obesity and differential improvement in depressive symptoms. Accordingly, further research in larger and more age-diverse samples, with designs that isolate pharmacologic mechanisms, will be necessary to clarify the clinical and mechanistic significance of these observed associations.

## Data Availability

The datasets used and/or analysed during the current study available from the corresponding author on reasonable request.
